# Long Term Complete Remission of Intraluminal Squamous Cell Carcinoma by Bronchoscopic Electrosurgery

**DOI:** 10.1155/DTE.4.9

**Published:** 1997

**Authors:** Peter M. J. M. De Vries, Pieter E. Postmus, Tom G. Sutedja

**Affiliations:** Department of Pulmonary Medicine Academic Hospital Vrije Universiteit Amsterdam The Netherlands

## Abstract

A patient is described with a synchronous intraluminal squamous cell carcinoma of the left upper lobe carina. He refused photodynamic therapy after sleeve lobectomy of the right upper lobe. The intraluminal tumor was treated with fiberoptic bronchoscopic electrosurgery. Complete remission was achieved, at the moment already for 36 months.


*Diagnostic and Therapeutic Endoscopy*, Vol. 4, pp. 9–11
Reprints available directly from the publisher
Photocopying permitted by license only

© 1997 OPA (Overseas Publishers Association)
Amsterdam B.V. Published in The Netherlands
by Harwood Academic Publishers
Printed in Singapore

### Long Term Complete Remission of Intraluminal Squamous Cell Carcinoma by Bronchoscopic Electrosurgery

PETER M.J.M. DE VRIES *, PIETER E. POSTMUS and TOM G. SUTEDJA

*Department of Pulmonary Medicine, Academic Hospital Vrije Universiteit, Amsterdam, The Netherlands*

*(Received 7 August 1996; In final form 23 January 1997)*

**A patient is described with a synchronous intraluminal squamous cell carcinoma of the left upper lobe carina. He refused photodynamic therapy after sleeve lobectomy of the right upper lobe. The intraluminal tumor was treated with fiberoptic bronchoscopic electrosurgery. Complete remission was achieved, at the moment already for 36 months.**

*Keywords:* Bronchoscopic electrosurgery, Curation, Squamous cell carcinoma

### INTRODUCTION

Tumor in the central airways can cause serious morbidity. Dyspnea, hemoptysis and pneumonia due to obstruction by the tumor can be palliated by bronchoscopic techniques [1] including electrosurgery as was recently shown in a pilot study [2]. The following case report is to show the curative potential of electrosurgery in a patient with intraluminal tumor.

### CASE REPORT

A 63-year-old man with a history of mild chronic obstructive pulmonary disease presented with worsening exercise-induced dyspnea. His medication included inhalation of salbutamol and budesonide. Breath sounds were increased at the apex of the anterior side of the right lung; expiration was normal. Inspiratory vital capacity was slightly decreased (4.2 liter, 72% of predicted) with moderate bronchoconstriction ($FEV_1/VC$ 0.55, 72% of predicted). A chest roentgenogram demonstrated atelectasis of the right upper lobe. Bronchoscopy and computed tomography revealed that this was due to a squamous cell carcinoma of the right upper lobe bronchus. At bronchoscopy a small elevation was seen at the carina of the left upper lobe (See Fig. 1) with a diameter of 5 mm. This synchronous squamous cell carcinoma was not visible at the CT scan, but was histologically proven. Sleeve resection of the right

* Corresponding author. Tel.: 31 20 4444 782. Fax: 31 20 4444 328.

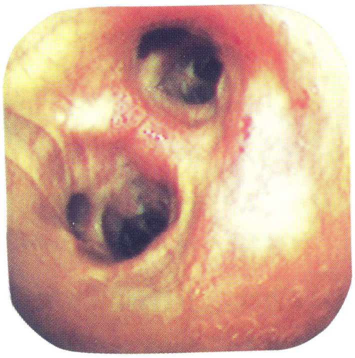

FIGURE 1 Small squamous cell carcinoma at the carina of the left upper lobe (diameter 5 mm).

upper lobe was performed, the margins being free of tumor ($T_1N_0M_0$).

Photodynamic therapy was proposed for the carcinoma in the left lung. However, the patient refused this due to the necessity to avoid exposure to any strong light, indoors or outdoors. Fiberoptic bronchoscopic electrosurgery was chosen as a treatment alternative. The procedure was performed under sedation with midazolam intravenously, according to the standards published recently [2]. Oxygen saturation was monitored by pulse oximetry and supplementary oxygen was given by nasal sprongs. The fiberoptic bronchoscope (Olympus$^R$, diameter 6 mm, diameter suction channel 2.6 mm) had an insulated and electrically grounded inner sheath. A flexible electrosurgery probe (diameter 1.2 mm) was used for coagulation with standard electrocautery equipment. Energy at 35 Watt was applied. The probe was gently pressed against the tumor and slowly advanced and retracted through the tumor during the coagulation procedure until coagulation appeared to be sufficient. The tip of the bronchoscope was then used to shear off necrotic tissue and repeated washing and suction was applied to clean up all tumor debris. A clean-up bronchoscopy appeared not to be necessary.

Repeated bronchoscopies 3, 6, 12, 18 and 36 months after the electro-surgical session did not show any sign of recurrence of tumor. (See Fig. 2)

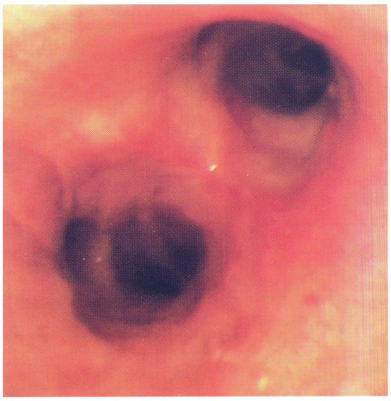

FIGURE 2 Carina of the left upper lobe 6 months after bronchoscopic electrosurgery.

### DISCUSSION

In patients with radiologically occult lung cancer, the standard treatment is surgery. In one study, even for these small bronchial cancers, a lobectomy was needed in 70 percent of the patients and a bilobectomy or a pneumonectomy in 30 percent [3]. The chance of getting a second lung cancer following the first resection was about 20 percent. Clearly, a curative treatment that does not result in loss of normal lung tissue would be desirable, particularly in patients with limited pulmonary reserve or with synchronous or metachronous tumors as presently described.

Technically speaking any bronchoscopic intervention is capable of eradicating small intraluminal tumors. A study of tissue necrosis after PDT, Nd-YAG laser, diathermy and cryosurgery indicated various necrotic depth up to several millimeters [1]. Bronchoscopic electrosurgery has not widely been used despite its advantages over PDT and Nd-YAG laser therapy. It is cost effective, easy and quick to perform, even under local anesthesia using standard electrosurgery equipment which is widely available in every hospital. The technique provides rapid pallation in patients with intraluminal tumor and a poor prognosis [2]. The only disadvantage is the unpredictable amount of necrosis. Nevertheless, this form of treatment might provide a cheap and safe alternative for curative endobronchial treatment of small intraluminal tumors as intraluminal early stage cancers are only several millimeters thick.

### *References*

[1] Sutedja, G. and Postmus, P.E. Bronchoscopic treatment in lung cancer. *A review. Lung Cancer* 1994; **11**: 1–17.

[2] Sutedja, G., Van Kralingen, K., Schramel, F. and Postmus, P.E. Fiberoptic bronchoscopic electrosurgery under local anesthesia for rapid palliation in patients with central airway malignancies. *A preliminary report. Thorax* 1994; **49**: 1243–6.

[3] Cortese, D.A., Pairolero, P.C., Bergstrahl, E.J., Woolner, L.B., Uhlenhopp, M.A. and Piehler, J.M. Roentgenographically occult lung cancer: a ten-year experience. *J. Thorac. Cardiovasc. Surg.* 1983; **86**: 373–80.

